# Role of Complement and Histones in Sepsis

**DOI:** 10.3389/fmed.2020.616957

**Published:** 2020-12-23

**Authors:** Firas S. Zetoune, Peter A. Ward

**Affiliations:** Department of Pathology, University of Michigan Medical School, Ann Arbor, MI, United States

**Keywords:** anaphylatoxins, histones, NLRP3 inflammasome, ROS, NETs, METs, C5a, C5b-9

## Abstract

The wide use of the mouse model of polymicrobial sepsis has provided important evidence for events occurring in infectious sepsis involving septic mice and septic humans. Nearly 100 clinical trials in humans with sepsis have been completed, yet there is no FDA-approved drug. Our studies of polymicrobial sepsis have highlighted the role of complement activation products (especially C5a anaphylatoxin and its receptors C5aR1 and C5aR2) in adverse effects of sepsis. During sepsis, the appearance of these complement products is followed by appearance of extracellular histones in plasma, which have powerful proinflammatory and prothrombotic activities that cause cell injury and multiorgan dysfunction in septic mice. Similar responses occur in septic humans. Histone appearance in plasma is related to complement activation and appearance of C5a and its interaction with its receptors. Development of the cardiomyopathy of sepsis also depends on C5a, C5a receptors and histones. Neutralization of C5a with antibody or absence of C5aR1 blocks appearance of extracellular histones and cell and organ failure in sepsis. Survival rates in septic mice are greatly improved after blockade of C5a with antibody. We also review the various strategies in sepsis that greatly reduce the development of life-threatening events of sepsis.

## Background

Development of infectious sepsis in humans often results in a series of events that can lead to death. In spite of nearly 100 clinical trials, no drug has been approved by the FDA for use in septic patients. The mouse model of infectious sepsis is polymicrobial sepsis produced by cecal ligation and puncture (CLP). This model has been widely used for four decades (1, 2) because it appears to mimic events occurring in septic humans. In the early phases of polymicrobial sepsis (first 1–3 days), there is hyperactivation of the innate immune system, releasing a flood of cytokines, chemokines, and extracellular histones, all of which cause injury to cells and organs (especially lungs, liver, spleen, heart, kidneys, and the central nervous system) (3–5). It is also established that aged septic mice (20–24 months of age) or septic humans (>60 years of age) have more severe sepsis and much greater lethality when compared to younger mice or humans, respectively (6–10). Reasons for these responses are not established, except, perhaps, for the fact that aged mice or humans have progressive impairments of the innate immune system (7, 11, 12). Beginning around 3 days after onset of sepsis, the innate immune system becomes progressively non-responsive, and immunosuppression sets in (5, 13). If the septic mouse or human is able to contain these responses, within about a week the innate immune system recovers, immunosuppression subsides and organ dysfunction is reversed, with return to the pre-septic state (14). It has recently been shown that, after discharge of patients from the hospital, there is evidence of residual medical problems in many individuals, especially the elderly (15, 16). For instance, nearly 50% of these individuals have a shortened life span over a 2-year period post-sepsis, resulting in a doubling of death rates when compared to a non-septic cohort. Obviously, there is a great deal to be learned about events in sepsis and how survival can be improved. Septic mice with various genetic manipulations appear to be critical resources for such studies. While the NIH/NIGMS has discouraged the use of mice for sepsis studies and is no longer engaged in supporting sepsis studies in humans, we have been able to identify mechanisms in septic mice that can be extrapolated to septic humans. Studies such as neutralization of histones or C5a have been shown to greatly improve survival in septic mice, employing interventions that could not be done in septic humans.

## Polymicrobial Sepsis Model and Blockade Strategies Targeting C5aRs Or Extracellular Histones

Most of the septic studies in mice use the polymicrobial sepsis model with CLP as the standard procedure (17, 18). The polymicrobial experimental model mimics sepsis in humans and is helpful for understanding the sepsis process in the human body (19, 20). In human sepsis, there is excessive C5a generation associated with inflammatory responses. In 2009, Xu et al. described the role of histones in mice with polymicrobial sepsis, endotoxemia or infusion of TNF (21). We have shown the harmful effects of C5a in septic mice, resulting in cardiomyopathy and cardiac dysfunction (22, 23). Blockade of C5a or its receptors (mainly C5aR1) significantly preserved heart dysfunction in septic mice. The same result developed in septic mice lacking C5a receptors (C5aR1 or C5aR2) (23). In these studies, cardiovascular performance was measured by ECHO/Doppler technology in mice before and 8 h after induction of CLP. Echocardiograms were also obtained before and after sepsis, according to the recommendations of the American Society of Echocardiography (23). Cardiac performance (especially isovolumic relaxation time) showed preserved levels of this parameter in the septic mice which lacked either C5a receptor (especially C5aR1) compared to the septic wild type mice (23).

Another strategy for reducing the harmful effects of sepsis was neutralization of extracellular histones (with clone BWA3 antibody targeting H2A/H4). Our functional studies showed remarkably reduced cardiac dysfunction in septic mice. Septic mice receiving this neutralizing antibody against histones showed preserved cardiac function as measured by Echo/Doppler studies (24).

## Phases of Polymicrobial Sepsis

It is now well-established in polymicrobial sepsis in mice that there is an early phase of robust activation of the innate immune system (1–3 days after CLP) during which time neutrophils (PMNs) and monocytes/macrophages release powerful proinflammatory mediators (TNF, IL-6, IL-1β, IL-17, etc.) in a “cytokine burst” that causes cell damage and multiorgan dysfunction, especially affecting kidneys, heart, liver, and other organs (13, 25, 26). As polymicrobial sepsis progresses, many of the proinflammatory responses are attenuated as immunosuppression develops at 3–7 days, resulting in reduced innate immune cellular responses which may compromise the natural protective responses that combat a variety of infectious agents (bacterial, fungal, viral) (5, 27, 28). If the protective immune responses are adequate, the inflammatory responses subside and mice are returned to health within 7 days, including recovery of organs from damage and reversion to the pre-sepsis state (14, 29).

The early phases of sepsis are associated with strong activation of the three complement pathways (classical, alternative, and lectin), generating a variety of strong proinflammatory peptides, especially anaphylatoxins C3a and C5a. C3a was originally identified by its ability to react with its receptor (C3aR), resulting in increased vascular permeability and smooth muscle contraction in a variety of tissues (3, 30, 31). The other anaphylatoxin, C5a, was recognized as a very powerful activator of PMNs and macrophages. Following its rapid binding to C5a receptors (C5aR1 and C5aR2), C5a·C5aR1 interaction causes activation of both PMNs and macrophages, the result being release of proinflammatory cytokines and chemokines, chemotaxis, generation of powerful and harmful oxygen-free radicals, and release of a variety of enzymes and lipid mediators that positively and negatively modify inflammatory responses (32–34).

### Sepsis-Induced Release of Histones and Mechanisms of Cell and Organ Damage Related to Histones

**Figure 1:**
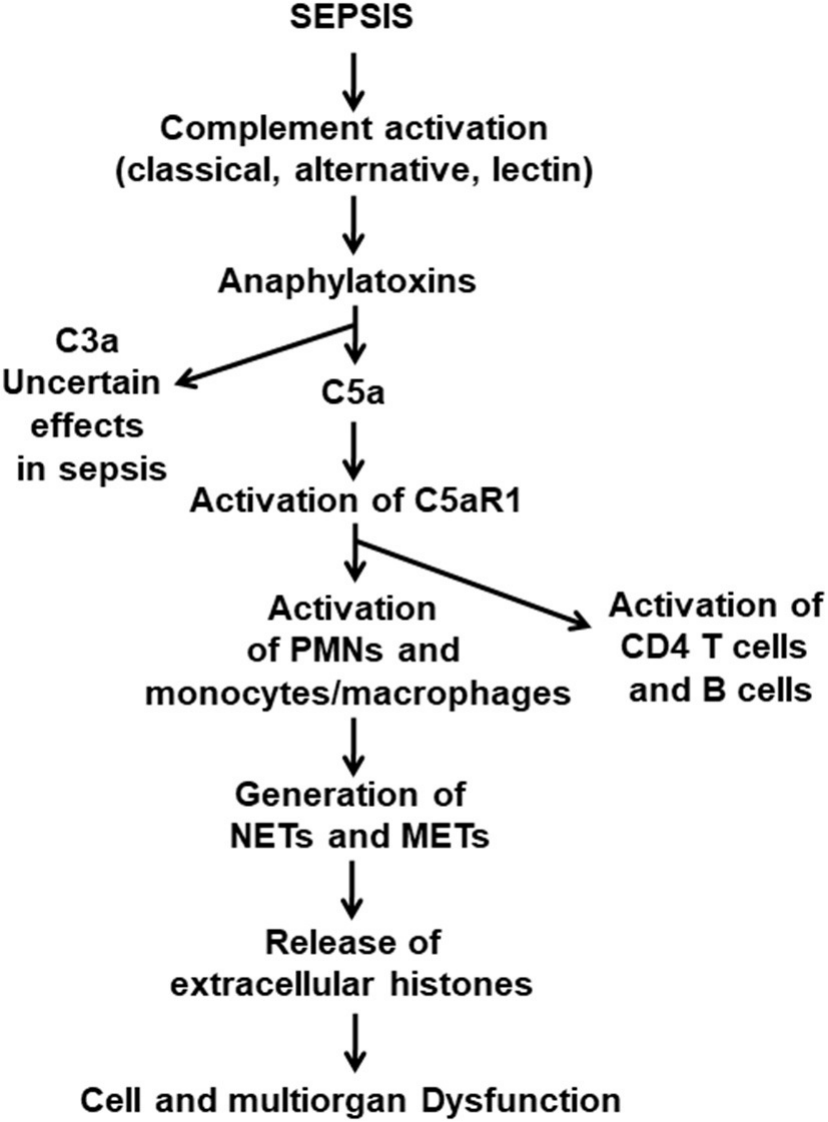
Pathophysiological consequences of sepsis. This figure is a composite of what is currently known about the pathophysiology of polymicrobial sepsis in mice. Onset of sepsis is associated with activation of the three pathways of complement activation, which results in appearance of the anaphylatoxins, C3a and C5a. Both anaphylatoxins have strong proinflammatory effects, and both interact with their receptors, C3aR and C5aR1 and C5aR2, especially on PMNs and macrophages. The literature indicates that the C5a axis of activation, involving C5aR1 and C5aR2, causes proinflammatory responses that are harmful to cells and organs. In addition, C5a interaction with C5aR1 activates PMNs and monocytes and macrophages which form NETs and METs that are strongly proinflammatory and prothrombotic. Similar events occur in septic humans.

It is commonly found that septic mice and septic humans develop activation of all three complement pathways (classical, alternative and lectin). As emphasized in Figure 1, early in sepsis in both mice and humans (over the first 24 h), via C5a·C5aR1 interactions, there is extensive cell and organ damage related to the surge in plasma of proinflammatory peptides. In addition, there is also an early (1–3 days) appearance in plasma of IL-1β associated with activation of the NLRP3 inflammasome in PMNs and macrophages (35, 36). TNF, IL-6, and the IL-17 family of factors are early proinflammatory peptides appearing in plasma (13, 37, 38). The role of complement and C5a and its receptors in activation of the NLRP3 inflammasome in septic mice has been described (39), as well as NLRP3-induced activation of CD4+ T cells (40). As time progresses, like the rest of the innate system, the NLRP3 inflammasome becomes functionally defective, resulting in diminished release of IL-1β. We have recently shown that the cardiac dysfunction and proinflammatory cytokines (including IL-1β) levels are significantly diminished in septic mice lacking NLRP3 (35). We also have shown the levels of extracellular histones were significantly lower in plasma from mice lacking NLRP3 or C5a receptors (24). It has been demonstrated that sepsis causes in PMNs the appearance of neutrophil extracellular traps (NETs) and in macrophages the appearance of macrophage extracellular traps (METs) (41–44). In both cases, these traps cause adherence of bacteria to the traps resulting in bacterial killing. At the same time, these traps contain a host of products from leukocyte granules, such as proteases, proinflammatory peptides as well as extracellular histones, which are strongly proinflammatory and prothrombotic, the composite resulting in extensive injury to cells and organs (41–43, 45).

### Biological Roles of Extracellular Histones in Sepsis

In the setting of sepsis, many of the proinflammatory and thrombogenic events affecting cells and organs can be attributed to extracellular histones which derive from activated PMNs and macrophages (41–44). We have shown numerous biological responses, including vascular leakage, buildup of PMNs and macrophages in organs, cell swelling and cytotoxic outcomes, LDH release, increased [Ca^2+^]i in cells, and release of cytokines and chemokines (46). We have also shown the biological activities related to purified individual histones (H1, H2A, H2B, H3, H4) (46). We have not yet determined to what extent various biochemical changes that develop in histone proteins (acetylation, methylation and ubiquination) affect the biological function of extracellular histones, but this likely happens in a manner that amplifies or reduces biological responses, induced by histones.

### Role of Toll-Like Receptors (TLRs) in Biological Responses in Sepsis

It has been known for some time that TLRs function as pattern recognition receptors and are critical for numerous responses of the innate immune system. TLRs are also involved in responses to LPS, with most evidence suggesting that TLR4 is critical for cell responses to LPS (47). We have recently shown that in septic mice, as early as 8 h after onset of sepsis, cardiomyocytes develop dysfunction and that such dysfunction can be linked to the role of complement activation, C5a and its receptors, as well as extracellular histones, causing cardiomyocyte dysfunction (24). We have seen decreased levels of [Ca^2+^]i in the cardiomyocytes from knockout (KO) of either TLR2 or TLR4 after infusion of the histones, suggesting these receptors may be linked to interactions with histones (24).

Regarding other TLRs, we have also shown that the full development of cardiac dysfunction developing in septic mice requires the presence of both TLR3 and TLR9 (48). Septic mice with KO of either TLR3 or TLR9 have attenuated cardiac dysfunction during sepsis, but the precise pathways responsible for such changes are not currently known. However, in recent studies, cardiomyocytes (from normal mice) exposed to the histone mix showed release of LDH and the septic hearts showed release of proinflammatory cytokines (TNF, IL-6, and IL-1β) that are greatly diminished in hearts from TLR3 or TLR9 KO mice (48). Obviously, much more data are needed in terms of roles of TLRs in sepsis.

### Protective Interventions in Septic Mice and Septic Humans

Based on the information in Figure 1, there are numerous interventions that protect mice from the damaging effects of infectious sepsis.

#### Complement Blockade

This has been done in septic mice using a variety of strategies, including KO of key components (49–51) for each of the three pathways (classical, alternative, lectin) of complement activation. The problem with such interventions is that there are extensive interactions involving activation products from each pathway reacting positively with all three pathways of complement. Accordingly, specific and limited pathway blockade of a single complement pathway does not often occur under such circumstances. The exception may be the C1 esterase inhibitor which blocks C1 of the classical pathway (52). This inhibitor has been used in human septic patients (53, 54), but the results have not been especially impressive. C1 esterase inhibitor is currently not used to treat septic humans (55).

#### Blockade Involving C5 or C5a Receptors

The absence (by KO) of C5 or its blockade with a mAb blocks both C5a and C5b-9 generation as obvious targets (49–51). Blockade of C5 with mAb has been shown to block inflammatory responses in rheumatoid arthritis (56, 57). The use of mAb to C5 has also been approved in patients with paroxysmal nocturnal hemoglobinuria (58) as well as patients with myasthenia gravis (59). Using KO mice, the absence of C5aR1 has been shown to block many of the harmful outcomes in septic mice (23, 51). KO of C5aR1 substantially reduces the harmful effects of sepsis in mice, reducing multiorgan injury and greatly improving survival after CLP (23, 51, 60). There are several companies that have developed small molecular weight inhibitors for C5aR1, but to date none have been approved for use in septic humans. The second C5a receptor, C5aR2 was originally described as a C5a “default receptor,” functioning as a compound that binds C5a, resulting in the absence of any signal-transduction response. There is conflicting information in the literature about the biological role of C5aR2 (formerly known as C5L2) (61, 62). Until this conflict is resolved, it seems unlikely that C5aR2 will be a target for pharmaceutical companies.

#### Blockade of Proinflammatory Peptides or Their Receptors in Sepsis

While blockade of TNF or its receptor, or mAb to C5 has been effective in patients with paroxysmal nocturnal hemoglobinuria or myasthenia gravis, similar interventions have not been successful in the setting of sepsis in mice or humans. This is probably due to the presence of numerous other proinflammatory peptides appearing in septic humans with similar biological activities.

#### Blockade of Histones in the Setting of Sepsis

As indicated above, it is now clear that extracellular histones derived from activated PMNs or macrophages in septic mice (or septic humans) appear in the plasma of septic mice or septic humans in the early phases of sepsis (1–3 days) in substantial amounts (25 μg/ml) as determined by immunological assays or by mass spectrometry (24, 63, 64). In septic mice, PMNs and macrophages appear to be the chief source of the extracellular histones. As indicated above, these histones are intensely proinflammatory and prothrombotic. Our studies indicate that appearance of extracellular histones in sepsis is complement and C5aR1-dependent. This suggests an alternative strategy to block appearance of extracellular histones in the setting of sepsis. However, much more information will be needed before interventions of histones in septic humans can be considered.

## Author Contributions

FZ and PW were involved in the work in mice with polymicrobial sepsis. Both participated in the writing and review of this report.

## Conflict of Interest

The authors declare that the research was conducted in the absence of any commercial or financial relationships that could be construed as a potential conflict of interest.
